# The Role of Hysteroscopy in the Diagnosis and Treatment of Adenomyosis

**DOI:** 10.1155/2017/2518396

**Published:** 2017-08-09

**Authors:** Attilio Di Spiezio Sardo, Gloria Calagna, Fabrizia Santangelo, Brunella Zizolfi, Vasilis Tanos, Antonino Perino, Rudy Leon De Wilde

**Affiliations:** ^1^Department of Public Health, School of Medicine, University of Naples “Federico II”, Naples, Italy; ^2^Obstetrics and Gynecology Unit, “Villa Sofia Cervello” Hospital, University of Palermo, Palermo, Italy; ^3^Department of Neuroscience, Reproductive Sciences and Dentistry, School of Medicine, University of Naples “Federico II”, Naples, Italy; ^4^European Academy for Gynaecological Surgery, Diestsevest, Leuven, Belgium; ^5^Department of Obstetrics, Gynecology, and Gynecology Oncology, Pius Hospital, Oldenburg, Germany

## Abstract

Uterine adenomyosis is a common gynecologic disorder in women of reproductive age, characterized by the presence of ectopic endometrial glands and stroma within the myometrium. Dysmenorrhea, abnormal uterine bleeding, chronic pelvic pain, and deep dyspareunia are common symptoms of this pathological condition. However, adenomyosis is often an incidental finding in specimens obtained from hysterectomy or uterine biopsies. The recent evolution of diagnostic imaging techniques, such as transvaginal sonography, hysterosalpingography, and magnetic resonance imaging, has contributed to improving accuracy in the identification of this pathology. Hysteroscopy offers the advantage of direct visualization of the uterine cavity while giving the option of collecting histological biopsy samples under visual control. Hysteroscopy is not a first-line treatment approach for adenomyosis and it represents a viable option only in selected cases of focal or diffuse “superficial” forms. During office hysteroscopy, it is possible to enucleate superficial focal adenomyomas or to evacuate cystic haemorrhagic lesions of less than 1.5 cm in diameter. Instead, resectoscopic treatment is indicated in cases of superficial adenomyotic nodules > 1.5 cm in size and for diffuse superficial adenomyosis. Finally, endometrial ablation may be performed with the additional removal of the underlying myometrium.

## 1. Introduction

Adenomyosis is defined as the presence of endometrial tissue (glands and stroma) within the myometrium; heterotopic endometrial tissue foci are associated with a variable degree of smooth muscle cell hyperplasia [1]. On the basis of myometrial invasion extension, it can be either* diffuse* or* focal*. In the diffuse type, endometrial glands and/or stroma are extensively intermingled with myometrial muscle fibers with an increase in uterine volume (proportionally correlated with the extent of lesions); focal adenomyosis is generally a single nodular aggregate located in the myometrium, which may have a histologic spectrum from mostly (“adenomyoma”) solid to mostly cystic (“adenomyotic cyst”) [2, 3].

The incidence rate of adenomyosis, generally defined on the basis of hysterectomy specimens, is extremely variable (ranging between 5% and 70%) mainly because of the lack of widely accepted criteria for histopathological diagnosis [4]. It is usually diagnosed in fertile-age women and possible risk factors appear to be pregnancy and previous operative procedures on the uterus.

The exact mechanisms of how adenomyosis develops are still unknown, but the current trend in thought is that adenomyosis or adenomyoma originate from the deep part of the endometrium that invaginates between the bundles of smooth muscle fibers of the myometrium itself, mainly after uterine traumatic events [5, 6]. Thus, uterine manipulations appear to be a crucial factor predisposing the invasion of endometrial cells into the myometrium [7]. Instead, for uterine adenomyotic cysts—a cystic structure lined with endometrial tissue and surrounded by myometrial tissue that, in most cases, contains haemorrhagic material—direct proliferation of metaplastic myometrial cells of endometrial tissue is supposed to be a possible pathogenetic mechanism, considering the common embryological origin from the Mullerian ducts of the endometrium and the subjacent myometrium [6, 8].

## 2. Symptoms

There are no symptoms or physical signs that are specific to adenomyosis. However, the cyclic bleeding of the ectopic endometrium resulting in irritation of surrounding tissue often leads to different nonspecific symptoms, including dysmenorrhea (starting at an early age around the time of menarche in the juvenile forms), abnormal uterine bleeding, chronic pelvic pain, and deep dyspareunia [8]. Dysmenorrhea tends to progressively increase and is resistant to therapy with analgesics or cyclic oral contraceptives.

The causative relationship between adenomyosis and subfertility has not been fully confirmed, and the incidence of subfertility in women with adenomyosis has not been defined [8, 9].

## 3. Diagnosis

Considering the poor specificity of preoperative clinical-based diagnosis, adenomyosis is often an incidental finding in specimens obtained from hysterectomy or uterine biopsies and/or percutaneous ultrasound-based biopsies. The recent evolution of diagnostic imaging techniques, such as transvaginal sonography (TVS), magnetic resonance imaging (MRI), and hysterosalpingography (HSG), has contributed to improving accuracy in the identification of this pathology, with an increasing number of cases described in adolescents and young adult women with untreatable dysmenorrhea [8, 10].

### 3.1. TVS

With TVS, an* adenomyomatous nodule *may be suspected when there is an oval, hypoechogenic, or hyperechogenic, noncapsulated area in the myometrial thickness, without the posterior cone of shadow and the hyperechoic margin (typical of uterine myomas), variable in diameter. Moreover, small cystic spaces filled with blood, which are rarely >5 mm, may be present [11]. Generally, colour Doppler sonography reveals rich vascularity, which does not circumscribe the lesion and presents an orthogonal orientation in relation to the endometrium [12, 13].

In rare cases, the lesion maybe be seen as a single cyst, with a diameter ≥ 1 cm, filled with a chocolate-brown-coloured fluid, namely* cystic adenomyosis*. It is considered an extremely rare variant of adenomyosis characterized by the presence of a haemorrhagic cyst resulting from menstrual bleeding in the ectopic endometrial glands [6].

Referring to the* diffuse* form, some sonographic characteristics can be helpful in making a differential diagnosis with uterine fibromyomatosis: increased volume uterus, asymmetry in the thickness of myometrial walls, myometrial linear layers, inhomogeneity, poorly defined endometrial-myometrial interface, and thickening of the subendometrial halo. Furthermore, the colour Doppler image shows typical accentuated vascularization with orthogonal orientation relative to the endometrium. Sometimes, there is a typical “Swiss cheese” appearance, presenting numerous, small, irregular cystic spaces (5–7 mm in diameter) within the myometrium [12, 14].

A TVS may also permit differentiation between surface and deep adenomyosis, which can be used to counsel the patient on the most appropriate therapeutic management: only the superficial variant is amenable to resectoscopic treatment [2, 14].

The application of 3D TVS to adenomyosis is a recent development and few articles have been published. As extensively described by Exacoustos et al., 3D ultrasound signs of adenomyosis are based on the evaluation of the junctional zone (JZ) (i.e., the inner myometrial layer immediately underlying the endometrium, composed of higher cellular density and a higher nuclear area compared to the outer myometrium) on the acquired volume of the uterus in order to obtain the coronal view; the JZ appears as a hypoechoic zone around the endometrium [15, 16].

Any irregularity in the JZ can be described in each location of the uterus; it could be visible and regular or, on the contrary, irregular, interrupted, not visible, or not assessable [15, 17]. For morphological descriptions of the JZ, objective parameters have been proposed, based on the MRI evaluation technique: thickness, regularity, and interruption of JZ [18]. In this way, 3D TVS, with the reconstruction of uterine anatomy in the coronal plane, provides a new view of the JZ, previously only well evaluated with MRI.

### 3.2. MRI

The JZ is easily seen with MRI, as a low signal intensity zone. The presence of adenomyosis is related to uncoordinated proliferation of these inner myometrial cells, resulting in focal or diffuse JZ hyperplasia: it can be seen on T2-weighted MRI as diffuse or focal thickening of the JZ.

As previously reported, the evaluation of the JZ in an MRI exam is based on three main parameters: thickness, regularity, and interruption of JZ.

Thickness must be measured at the thinnest (JZmin) and thickest (JZmax) part, at the anterior and posterior wall in the sagittal slices. Diffuse adenomyosis is diagnosed given a JZmax of 15 mm. If the JZ is 12–15 mm in thickness, a diagnosis of adenomyosis arises only in the presence of additional criteria (such as loss of smooth appearance of the JZ, poorly circumscribed foci within the myometrium with high or low intensity) [14, 19]. In cases of focal adenomyosis, T2-weighted sequences show a decreased subendometrial signal intensity (consistent with the presence of necrotic tissue) with blurred margins surrounding the lesion [14].

An additional proposed parameter is the difference between JZmax and JZmin (JZdif), calculated for the anterior or posterior border. Any eventual JZ irregularity can be measured as JZdif, representing an additional characteristic of adenomyosis [20].

Diagnostic MRI cluster of cystic adenomyosis relies on the detection of a complex cystic lesion that is usually located within the myometrium, with hyperintense T1-w and intermediate to hyperintense T2-w signal contents suggesting haemorrhagic and/or proteinaceous products. In addition, the presence of surrounding T2-hypointense tissue is indicative of reactive myometrial hypertrophy, and/or thin rim cystic wall may show hypointense T1-w and T2-w signal, suggestive of the presence of hemosiderin due to endometrial sloughing [21].

### 3.3. HSG

Occasionally, HSG is of diagnostic value, identifying one or more of the following features: “*lollipop-like” *diverticula (direct sign), dilated tubes (indirect sign), and prominent spicules (indirect sign).

### 3.4. Hysteroscopy

Hysteroscopy offers the advantage of direct visualization of the uterine cavity while giving the option of collecting histological biopsy samples under visual control [22, 23].

**Figure 1:**
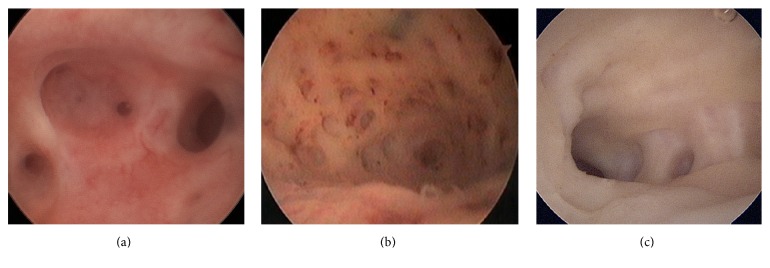
Hysteroscopic view of irregular endometrial mucosa due to the presence of tiny openings on the endometrial surface. These images suggest the hypothesis of adenomyosis.

**Figure 2:**
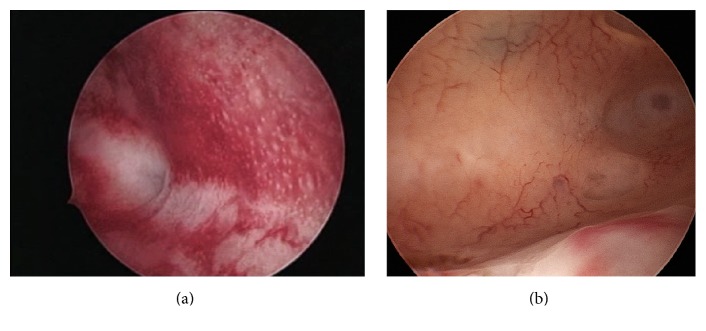
Hysteroscopic image suggestive of adenomyosis under hysteroscopic examination and then confirmed at histological exam after hysterectomy. (a) The typical endometrial “Strawberry” pattern with signs of hyperemia and areas appearing bright red harbouring white central dots. Fundal, irregular vascularization, small subendometrial haemorrhagic cyst.

**Figure 3:**
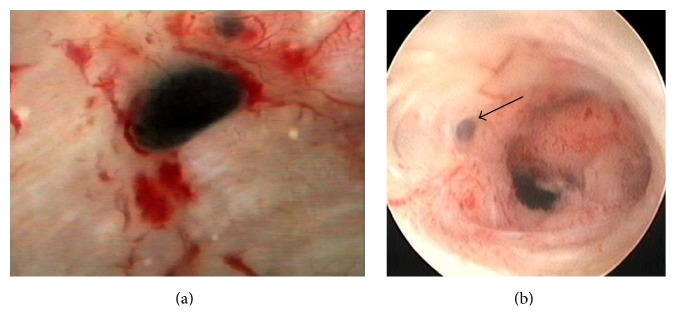
Hysteroscopic images of small haemorrhagic foci assuming a chocolate brown colour, suggestive of adenomyosis (diagnosis was confirmed at histological exam on target-eye biopsies).

However, diagnostic hysteroscopy cannot establish a definitive diagnosis of adenomyosis, considering that its field of vision is restricted to the endometrial surface layer. The following aspects are generally indicative of the pathological condition:

- irregular endometrium with tiny openings seen on the endometrial surface (Figure 1);
- pronounced hypervascularization;
- an endometrial “strawberry” pattern (Figure 2(a));
- fibrous cystic appearance of intrauterine lesions (following 3–5 episodes of intramyometrial haemorrhage) (Figure 2(b));
- haemorrhagic cystic lesions assuming a dark blue or chocolate brown appearance (Figure 3) [24].

In patients with adenomyosis, hysteroscopy reveals an irregular endometrial vascular distribution pattern, both during the proliferative and secretory phase. Moreover, it allows obtaining biopsy samples from the endometrium and underlying myometrium using mechanical instruments (biopsy or grasping forceps, scissors) or bipolar electrodes [24].

The traditional technique to assess the extent of adenomyosis infiltration is biopsy sampling performed using a resectoscope with a diathermic loop, to perform the resection of the endomyometrial layer concerned. In order to obtain an adequate biopsy with this approach, it is first of all necessary to have a specimen including both the endometrium and the underlying myometrium layer and then take a second biopsy deeper into the dent left behind by the first, including only myometrial tissue [15, 16]. Generally, on resectoscopic biopsy sampling, the following three clues can be strongly suggestive of adenomyosis: (1) irregular subendometrial myometrium (spiral and/or fibrotic); (2) contortion of normal myometrial architecture noticeable during resection; (3) presence of intramural endometriomas.

Recently, Gordts et al. described the use of a new gliding system, the* Trophy*°* Hysteroscope (Karl Storz, Germany)* that offers the possibility of changing from a diagnostic 2.9-mm hysteroscope to an operative 4.4-mm scope without the need to remove the hysteroscope [8]. 5-Fr instruments are used for dissection/coagulation through the operative channel. With the application of a new device, the* Utero-Spirotome*, it is possible to have “direct and frontal” tissue harvest, allowing the biopsy of endomyometrial layers.

*Spirotome *operates with two devices: the receiving needle with a cutting helix at the distal end and a cutting cannula as an outer sheet; the correct direction and position of the helix point must be under continuous ultrasonographic imaging and hysteroscopic control. In this way, the Spirotome can be directed towards any intramural localized lesion such as cystic adenomyosis, thus creating a visible hysteroscopic channel that allows access to the cystic cavity. Thanks to this device, the hysteroscopy can offer an alternative access to cystic adenomyosis, producing minimal tissue damage [8].

## 4. Treatment

Considering the relevant technical progress seen in recent years and the increasing rate of preoperative diagnosis of adenomyosis, it is currently possible to perform a “tailored” treatment for any patient, based on the several available medical and surgical options.

The factors to be taken into consideration in order to choose the correct therapeutic strategy are patient age, desire for a future pregnancy, symptoms, and coexisting pelvic diseases.

Medical approach to adenomyosis disease is based on its hormone dependent nature and on its similarities to endometriosis: in this sense, effective therapies for endometriosis can potentially be adequate for adenomyosis. Generally used medical treatments for adenomyosis include gonadotropin-releasing hormone agonists (GnRH-a), levonorgestrel-releasing intrauterine device (LNG-IUD), oral contraceptive combined pill, progestogens, and danazol [25–27]. The principal limit of these medical options is that they induce regression but not eradication of the pathology, with symptom recurrence after drug discontinuation.

Regarding surgical treatment, a critical question is whether to remove or preserve the uterus. Hysterectomy remains the standard treatment for symptomatic adenomyosis for patients who do not desire a future pregnancy and who accept the operation. Hysterectomy can be performed by laparoscopy or laparotomy or vaginally, based on uterine size, parity, and surgeon's experience.

In the cases where women decline hysterectomy or wish to pursue future pregnancy and the surgical approach is chosen, it is necessary to accurately define the characteristics of the adenomyosis and consequently the “way” to access the lesions for conservative treatment. The concept of conservative “uterine-sparing” surgery (either performed by laparoscopy/laparotomy or hysteroscopy) for adenomyosis is increasing as fertility preservation and quality-of-life improvement can be achieved in this group of patients [28].

The surgical technique for excision of focal adenomyosis, by laparotomy or laparoscopy, is similar to myomectomy in many technical aspects, although it can be more difficult because adenomyosis generally lacks a cleavage plane. When the adenomyotic lesion can be clearly preoperatively defined (by TVS or MRI) laparoscopy is a feasible technique, and laparoscopic suturing presents no more difficulty compared with suturing after myomectomy for a skilled surgeon [29].

Diffuse adenomyosis typically involves the myometrium in an irregular and massive way, characterized by lesions with unclear borders, so much so that complete excision of adenomyotic tissue is not possible, with obligate loss of healthy myometrium. In these cases, the laparotomic approach should be chosen, because digital palpation of the uterus is necessary to delineate the involved areas, limiting the excision of healthy myometrium. Preservation of at least 1–1.5 cm of myometrial thickness is needed for uterine reconstruction, which can be particularly challenging after an extensive excision. Multiple layers of interrupted sutures are preferred for good repair and better obstetrical outcome [30].

Finally, preliminary results of “nonexcisional” conservative techniques are described in recent literature, such as laparoscopic electrocoagulation of the myometrium, laparoscopic uterine artery ligation, ablation of focal adenomyosis with high frequency ultrasound (HIFU), alcohol instillation under ultrasound guidance for the treatment of cystic adenomyosis, radiofrequency ablation of focal adenomyosis, and balloon thermoablation for diffuse adenomyosis [31].

Nevertheless, data supporting these types of intervention are still suboptimal, and prospective, comparative studies are needed.

## 5. Hysteroscopic Treatment

Hysteroscopy is not a first-line treatment modality for women with adenomyosis and represents a viable option only in select cases of the focal or diffuse superficial subtype.

### 5.1. Office Hysteroscopy

**Figure 4:**
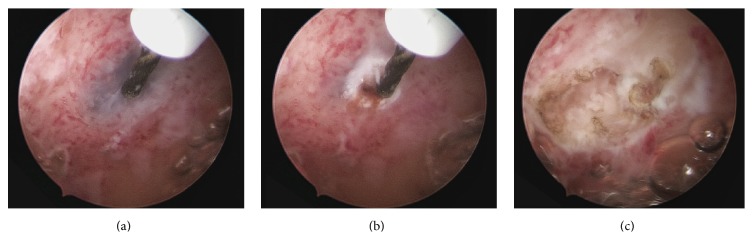
Evacuation of hypothesized superficial adenomyotic cysts with a 5-Fr bipolar electrode (KARL STORZ, Germany). Panoramic image of the small cystic lesion (a). Incision and drainage of the cystic lesion (b-c).

**Figure 5:**
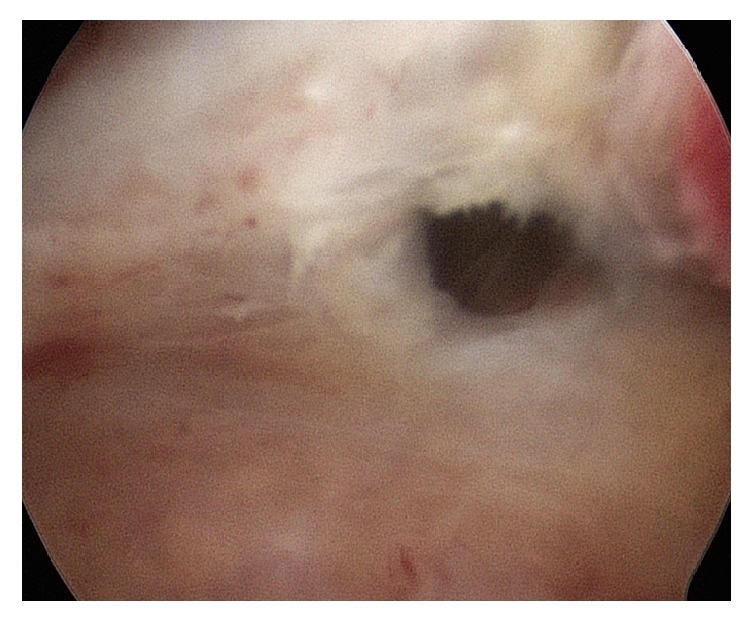
Hysteroscopic image of cystic adenomyotic lesion at the fundus of uterus affected by diffuse adenomyosis (diagnosis was confirmed at histological exam on target-eye biopsies), after incision with bipolar electrodes.

It is possible to enucleate superficial focal adenomyomas or to evacuate cystic haemorrhagic lesions of less than 1.5 cm in diameter, using mechanical instruments and/or bipolar electrodes (Figure 4). This treatment is feasible only when the lesions are directly recognizable at hysteroscopy as they bulge into the endometrial cavity, thus favouring a minimally invasive dissection (Figure 5).

The traditional technique adopted in the ambulatory setting is the same used for enucleation of submucosal myomas with an intramural component, even though the procedure involves a considerable element of precautionary exploration due to the lack of a distinct cleavage plane required for adequate identification of healthy myometrial tissue [8, 22].

Moreover, for cystic lesions localized deeper in the intramural portion (defined as* subtype A* by Brosens et al. in a recent review on the topic) [10], the Spirotome is a very useful innovation; under ultrasound guidance, access is gained to intramural cystic lesions without visible intracavitary components. The device creates a channel and provides hysteroscopic access to the cystic structure. Treatment by resection or bipolar coagulation can then be performed [8].

### 5.2. Resectoscopic Treatment

This is indicated in cases of superficial adenomyotic nodules > 1.5 cm in size and for diffuse superficial adenomyosis. Endometrial ablation may be performed with the additional removal of the underlying myometrium* (endomyometrectomy)* mainly in women not desiring future pregnancy.

*Deep diffuse* adenomyosis, instead, is not manageable with the hysteroscopic approach [32]. Some authors have demonstrated that, given a deep adenomyosis, resectoscopic treatment not only fails to reduce symptoms, but may even have adverse effects in that it masks the onset of deep adenomyosis developing below the endomyometrial scar tissue, which may consecutively be prone to malignant transformation.

To treat* focal *adenomyosis, the technique of adenomyomectomy has yet to be exhaustively defined. Tissue protruding into the uterine cavity is incised, evacuated, and resected (by slicing) using a resectoscope with a cutting loop. In cases of deeply implanted lesions, the nodule may first be mobilized using various techniques that cause it to migrate into the uterine cavity. These techniques have already been described for the treatment of a submucosal myoma with an intramural component, which is serially resected with a cutting loop until it can be completely removed. The surgical procedure is completed by coagulating the implantation base of the lesion.

The goal of surgery is to remove all adenomyotic tissue without damage to healthy surrounding myometrial fibers. However, the lack of a distinct cleavage plane indicating the normal myometrial tissue can make the procedure quite challenging [33, 34].

*Superficial diffuse* adenomyosis may be treated with endomyometrial ablation (endomyometrectomy). The technique differs from the classical method of endometrial ablation as resection is not limited to the endometrium and the first 2-3 mm of myometrium; rather, the operator proceeds with continued slicing of the myometrial layer below, until healthy myometrium is visualized, and concludes the procedure by coagulation of endometrial residues. The procedure is accomplished using 3-mm or 5-mm straight loops for ablation of the fundus and cornual recesses, as well as classic cutting loops for ablation of uterine walls.

The level of intramural extension of pathology is correlated with the technical difficulty and risks of the procedure. In the case of persistence and/or recurrence of disease, a second-stage surgical procedure may be performed.

However, meticulous care must be paid to the thickness of the myometrium between the outer margin of adenomyosis and the uterine serous surface, evaluated by ultrasound. Endomyometrectomy may give rise to dissemination and proliferation of ectopic endometrial cells, promoting progression of the pathology and “de novo” adenomyosis.

Adjunct to surgery, or as an alternative option, local medical therapy by application of a levonorgestrel-releasing IUD may be chosen. The continuous controlled release of LNG, directly at uterine mucosal level, may induce regression of adenomyotic lesions along with relief of pain symptoms [35].

## 6. Conclusions

Hysteroscopy has revolutionized the way to approach adenomyosis, in terms of both diagnosis and therapy.

Traditionally, adenomyosis has been diagnosed by the pathologist in hysterectomy specimens. Thanks to the advent of high definition noninvasive methods (TVS and MRI) diagnosis comes earlier and on a large scale. Moreover, the possibility of presurgical diagnosis allows an individualized therapeutic approach based on the individual needs of the patient.

The introduction and successive diffusion of office hysteroscopy has greatly enriched the diagnostic phase of this pathology, adding all the information regarding the endometrial surface of the affected uteri. Furthermore, hysteroscopy allows obtaining target-eye biopsies (also guided by sonographic control), which can definitively confirm the endoscopic diagnosis.

Hysterectomy remains the standard treatment for symptomatic adenomyosis if fertility is not an issue. In the cases of women who refuse hysterectomy or desire a future pregnancy, the decision is more complex. The use of medical therapies is mainly based on the observation that the disease is hormone dependent and on the similarities to endometriosis. Regarding surgery, the approach to focal deep adenomyosis by laparoscopy or laparotomy reflects the similarity of this pathology to a leiomyoma, although the technique for adenomyomectomy can be more challenging.

Finally, operative hysteroscopy (either with miniaturized instruments or standard resectoscope) may be indicated in cases of superficial adenomyotic nodules and for diffuse superficial adenomyosis.

Conservative uterine-sparing treatments of adenomyosis appear to be feasible and efficacious. An improvement of dysmenorrhea and menorrhagia is achieved in more than 81% and 50% of the patients, respectively [31]. On the other hand, data supporting this type of intervention for improving pregnancy outcome are still suboptimal and need to be confirmed by well-conducted studies, where many potential confounding factors of infertility should be adequately assessed [29, 36, 37].
